# Identification of a panel of tumor-associated antigens from breast carcinoma cell lines, solid tumors and testis cDNA libraries displayed on lambda phage

**DOI:** 10.1186/1471-2407-4-78

**Published:** 2004-11-12

**Authors:** Emiliano Pavoni, Paola Vaccaro, Andrea Pucci, Giorgia Monteriù, Elisa Beghetto, Stefano Barca, Maria Luisa Dupuis, Adolfo De Pasquale Ceratti, Antonio Lugini, Maurizio Cianfriglia, Enrico Cortesi, Franco Felici, Olga Minenkova

**Affiliations:** 1 Kenton Labs, c/o Sigma Tau, Pomezia (Rome), 00040, Italy; 2 Laboratorio di Immunologia, Reparto Immunologia dei Tumori, Istituto Superiore di Sanità, Roma, 00100, Italy; 3 Oncologia Medica, Dipartimento di Medicina Sperimentale e Patologia, Facoltà di Medicina e Chirurgia, Università di Roma "La Sapienza", Rome, 00100, Italy; 4 Dipartimento di Scienze Microbiologiche, Genetiche e Molecolari, Università di Messina, 98100, Italy

## Abstract

**Background:**

Tumor-associated antigens recognized by humoral effectors of the immune system are a very attractive target for human cancer diagnostics and therapy. Recent advances in molecular techniques have led to molecular definition of immunogenic tumor proteins based on their reactivity with autologous patient sera (SEREX).

**Methods:**

Several high complexity phage-displayed cDNA libraries from breast carcinomas, human testis and breast carcinoma cell lines MCF-7, MDA-MB-468 were constructed. The cDNAs were expressed in the libraries as fusion to bacteriophage lambda protein D. Lambda-displayed libraries were efficiently screened with sera from patients with breast cancer.

**Results:**

A panel of 21 clones representing 18 different antigens, including eight proteins of unknown function, was identified. Three of these antigens (T7-1, T11-3 and T11-9) were found to be overexpressed in tumors as compared to normal breast. A serological analysis of the 21 different antigens revealed a strong cancer-related profile for at least five clones (T6-2, T6-7, T7-1, T9-21 and T9-27).

**Conclusions:**

Preliminary results indicate that patient serum reactivity against five of the antigens is associated with tumor disease. The novel T7-1 antigen, which is overexpressed in breast tumors and recognized specifically by breast cancer patient sera, is potentially useful in cancer diagnosis.

## Background

A recent development in tumor immunology is based on the idea that the immune system can distinguish between normal and tumor tissues. Various studies suggest that both the cellular and humoral components of the immune system are able to recognize tumors (see review of Lake et al.) [1]. The presence of natural antibodies against cancer cells in peripheral blood of tumor patients probably plays a protective role against tumor development. The latest advances in molecular techniques further support the existence of natural antibodies against cancer antigens. The SEREX approach, based on the serological screening of cDNA expression libraries generated from tumor tissues of various origin, led to the molecular definition of immunogenic tumor proteins (tumor-associated antigens, TAAs) based on their reactivity with autologous patient sera [2]. This type of screening of a cDNA expression library is quite a laborious procedure requiring the preparation of a large number of membrane filters blotted with bacteriophage plaques, which are then screened with sera from cancer patients, usually available in limited quantity. In contrast to SEREX, phage display strategy is based on the selection and enrichment of antigens displayed on the phage surface. A physical link between a displayed fusion protein and the DNA encoding for it makes this phage target selectable through affinity purification. Phage display technology has been successfully applied to the screening of cDNA libraries from different tumors using the antibody repertoire of cancer patients [3-6]. In these experiments different phage display systems were used. Some of the authors used the C-terminus of a filamentous phage minor protein pVI for expression of cDNA libraries from breast cancer cell lines T47D and MCF-7 [3] and from colorectal cancer cell line HT-29 [5]. However, the filamentous phage display system imposes some biological bias for the expression and display of fusion proteins, since a filamentous phage-based library displays only those recombinant proteins able to pass through the inner bacterial membrane during filamentous phage assembly. To overcome this potential problem the lytic bacteriophages T7 [4] and λ [6] were used. By using these latter systems, the phage capsid is assembled in the cytoplasm of bacteria and mature phage particles are released by cell lysis. For example, Hansen and co-workers in their studies screened a commercially available (Novagen) human breast cancer cDNA library cloned in T7 vector [4], identifying positive clones.

Usually cDNA libraries are generated as C-terminal fusions. When such a library is panned on a serum, the presence of a complex antibody repertoire gives to out-of-frame or antisense-derived cross-reactive short peptide sequences a good chance of being enriched. In our previous work [6] we designed a new-concept lambda vector for the display of cDNA-encoded protein fragments as fusion to the N-terminus of bacteriophage gpD, allowing us to overcome this obstacle. In this vector, phage clones display a given protein fragment on the phage surface only when the insert's correct reading frame matches that of gpD. The size of the cloned DNA fragments in our libraries was adjusted to an average of 200–300 base pairs, which is of a size reasonably sufficient to potentially encode for a protein domain. The vast majority of out-of-frame sequences of the above-mentioned length most probably contains at least one in-frame stop codon. Thus, these inserts are not expressed as D fusion, are consequently not displayed on the phage surface and cannot be selected. In such cases, phage capsid contains only wt gpD encoded by lambda genome *D *gene. The N-terminal display system greatly reduces the selection of artifactual peptides, in comparison with a C-terminal fusion library displayed on lambda ([7] and our unpublished data).

By employing the SEREX approach numerous tumor antigens from different human neoplasms were identified [8,9]. Analysis of TAA expression in tumor samples and normal tissue led to the identification of a group, called cancer/testis antigens. Members belonging to this family are aberrantly expressed in human cancers and only in normal testis, but not in other normal tissue. For this reason, in addition to tumor samples and tumor cell lines, testicular cDNA libraries are also a convenient source of antigens which can be identified by screening with sera derived from tumor patients [10,11].

In the present work we report the construction of lambda-displayed cDNA libraries from breast cancer cell lines MCF-7, MDA-MB-468, from human breast carcinomas and from human testis, generated according to an improved protocol. These libraries were screened by using sera from breast cancer patients. The list of 21 identified antigens contains eight proteins with still unknown functions. Three of the genes (T7-1, T11-3 and T11-9) were found to be overexpressed in tumors as compared to normal breast. Recognition by human sera of five of the selected antigens (T6-2, T6-7, T7-1, T9-21 and T9-27) was associated with cancer diagnosis.

## Methods

### Tissue and serum samples

Specimens of breast carcinoma and autologous sera from breast cancer patients (B81-B96) were obtained from M. G. Vannini Hospital, Rome. A panel of human sera from breast cancer patients B1-B20, B36-B80 was provided by the Division of Medical Oncology, Federico II University of Naples. All the human biological samples were obtained through informed consent.

### Construction of λKM8, λKM10 vectors

**Figure 1:**
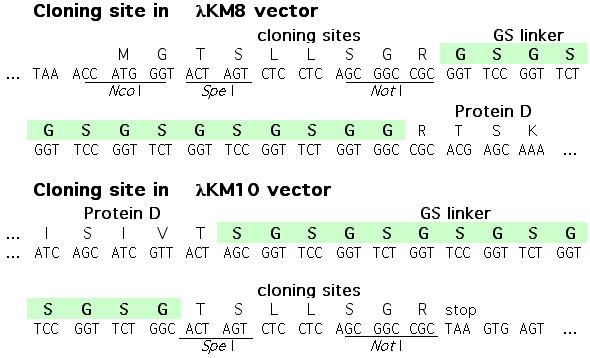
Cloning sites of λKM8 and λKM10 vectors.

λKM8 was constructed by cloning the oligonucleotide duplex  KM46 5’-CTAGTCTCCTCAGCGGCCGCGGTTCCGGTTCTGGTTCCGGTTCTGGTTCCGGTTCTGGT-3’ and KM47 5’-GGCCACCAGAACCGGAACCAGAACCGGAACCAGAACCGGAACCGCGGCCGCTGAGGAGA-3’ into *Spe*I, *Not*I sites at the 5'-end of the *D *gene in λKM4 vector [6]. The resulting vector λKM8 maintains the unique *Spe*I and *Not*I sites and encodes for a GS linker between the fusion site and gpD, Figure 1.

The plasmid pKM7 is a derivative of pKM3 [6], which was obtained by cloning of the oligonucleotide duplex K52  5’-GACCGCGTTTGCCGGAACGGCAATCAGCATCGTTACTAGTTTATTAAGCGGCCGCTAAGTGAGTG-3’ K53  5’-AATTCACTCACTTAGCGGCCGCTTAATAAACTAGTAACGATGCTGATTGCCGTTCCGGCAAACGCG-3’ into pKM3 previously digested with *Rsr*II and *EcoR*I restriction enzymes. pKM7 was digested with *Spe*I and *Not*I to obtain pKM9, by direct cloning of the oligonucleotide duplex KM48 5’-CTAGCGGTTCCGGTTCTGGTTCCGGTTCTGGTTCCGGTTCTGGCACTAGTCTCCTCAGC-3’ and KM49 5’-GGCCGCTGAGGAGACTAGTGCCAGAACCGGAACCAGAACCGGAACCAGAACCGGAACCG-3’. λKM10 was constructed by cloning pKM9, which was linearized by digestion with *Xba*I restriction enzyme, into the *Xba*I site of λ*Dam15imm21nin5 *[12]. The resulting vector λKM10 bears unique *Spe*I, *Not*I sites at the 3'-end of the *D *gene and encodes for a flexible GS linker between gpD and the cloned protein fragment, Figure 1.

### RNA extraction

mRNA from breast carcinoma cell lines MCF-7 and MDA-MB-468 was isolated in a single step by QuickPrep Micro mRNA Purification Kit (Amersham Pharmacia Biotech, UK) according to manufacturer's instructions.

Tumor samples from breast carcinoma patients were obtained as surgical specimens and immediately frozen in liquid nitrogen. Total RNA was prepared by Total RNA Isolation System (Promega, Madison, WI) and purified to Poly A+ RNA using PolyATract mRNA Isolation Systems (Promega).

Total RNA from normal testis was purchased from Genpak, UK (# 061023). Total RNA from normal breast (pool of 3) was purchased from Stratagene, La Jolla, CA (# 735044).

### cDNA library construction

From 1 to 5 μg of the purified poly(A)^+ ^RNA from cell lines or human tissues were used to synthesize cDNA by random priming, using TimeSaver cDNA Synthesis Kit (Amersham Pharmacia Biotech, Piscataway, NJ, USA). RNasin Ribonuclease Inhibitor (Promega) was added to first-strand synthesis reaction.

A mixture of the following oligonucleotides (130 pmol): K64 (5'-GCGGCCGCTGGNNNNNNNNN-3'), K79 (5'-GCGGCCGCTGGCNNNNNNNNN-3'), and K81 (5'-GCGGCCGCTGGCANNNNNNNNN-3') was used for priming. They all carry a *Not*I site (underlined) at their 5' end, and a random sequence of nine nucleotides at their 3' end, positioned in the three possible reading frames. The second strand was synthesized by nick translation according to the manufacturer's instructions.

One hundred ng of ds cDNA were randomly primed with 25 pmol of oligonucleotide K56 (5'-GGCCGGCCAACNNNNNNNNN-3'), constituted by a constant sequence at the 5' end, and a random 3'sequence. The reaction mixture was purified by QIAgen QIAquick columns.

Approximately 0,2 ng of the above randomly primed ds cDNA was amplified by PCR with biotinylated primers: K59 (bio-5'-GCACTAGTGGCCGGCCAAC-3'), K60 (bio-5'-GCACTAGTCGGCCGGCCAAC-3'), K61 (bio-5'-GCACTAGTCGGGCCGGCCAAC-3') and K65 (bio-5'-GGAGGCTCGAGCGGCCGCTGG-3'). K59, K60 and K61 carry the same constant sequence of K56 positioned in the three possible frames with respect to a *Spe*I site (underlined) allowing directional cloning. K65 carries a *Not*I site (underlined), that anneals to the 5' end of the reverse strand of cDNA.

PCR product was purified with QIAquick PCR purification kit (QIAGEN, Germany), filtered by Microcon-100 columns (Millipore, Bedford, MA) to reduce the number of small fragments and additionally fractionated by 6% PAGE. DNA smear, corresponding to 300–1000 base-pair fragments, was cut and eluted from gel according to standard procedure [13].

After digestion with *Spe*I and *Not*I enzymes, in order to remove the biotinylated extremities and uncut fragments, a 20-minute incubation with streptavidin M-280 Dynabeads (DYNAL, Norway) was performed. After additional filtration on Microcon-100 the insert was cloned in λKM8 or λKM10 vectors.

The vector was digested with *Spe*I, *Not*I enzymes and dephosphorylated. For each library 5 ligation mixtures, each one containing 0.5 μg of vector and about 3 ng of insert, were performed. After overnight incubation at 4°C the ligation mixtures were packaged *in vitro *by lambda packaging extract (Stratagene, La Jolla, CA). BB4 cells were infected by lambda and plated in top-agar on 100 (15 cm) NZY plates. After overnight incubation phages were eluted from the plates with SM buffer, purified, PEG/NaCl precipitated [13] and stored at -80°C in SM buffer, 7% DMSO.

### Affinity selection

Two μl of human serum were preincubated with 10 μl of BB4 bacterial extract and 10 μl of UV-killed lambda phage in 1 ml of blocking buffer (3% BSA, 1X PBS, 10 mM MgSO_4_, 1% Triton) for 30 minutes at 37°C under gentle agitation. 10^10 ^pfu of lambda library were then added to the preincubated mixture for a further incubation of 1 hr. Magnetic beads (100 μl), linked to Protein A (Dynabeads Protein-A, Dynal, Norway) were washed twice with the blocking solution. Mixture of library with serum was incubated with the beads for 10 min at RT under agitation. The beads were washed 10 times with 1 ml of washing solution (1X PBS, 1% Triton, 10 mM MgSO_4_). The bound phages were recovered by infection of 600 μl BB4 cells added directly to the beads. After a 20-minute incubation 10 ml of molten NZY-top agar (48°C) was added to the mixture of beads with infected cells and immediately poured onto NZY plates (15 cm). Next day the phage particles were harvested by incubation of the plates under agitation with 15 ml of SM buffer for 4 hours at 4°C. The phage particles were purified by PEG/NaCl precipitation and stored in 1/10 of initial volume of SM with 0.05% NaN_3 _at 4°C.

### Analysis of gene expression by PCR

Five hundred ng of poly(A)^+ ^RNA from breast carcinomas or normal tissue were used to synthesize full-length cDNA by SMART cDNA library construction kit (Clontech, Palo Alto, CA). For maximum sensitivity specific primers for the different genes were designed to amplify sequences located near the 3' end of gene's transcript. Twenty-five cycles of PCR were performed from 1 μl of each cDNA template, normalized through PCR amplification of the β-actin gene.

## Results

### Construction of the libraries

**Figure 2:**
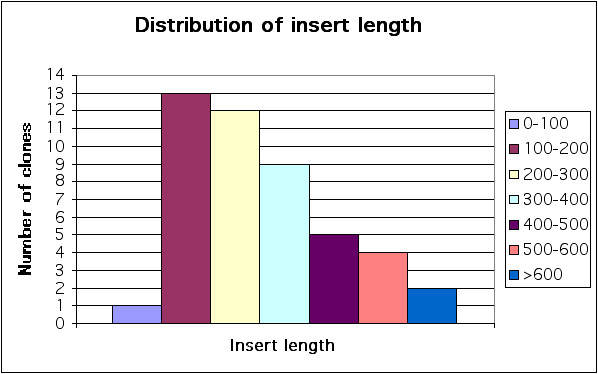
**Insert length distribution. **Forty-eight random clones from T5 library were amplified by PCR using a couple of primers on the sides of insert. Size of inserts was calculated according to their electrophoretic mobility in 3% agarose gel.

Lambda libraries were constructed by directional cloning of randomly primed cDNA from human breast carcinoma cell lines MCF-7 and MDA-MB-468, from human breast carcinomas or from human testis into the phage display vector λKM8 to generate fusions with the N-terminus of gpD (see list of libraries in Table 1). Only library T6 was built like C-terminal fusions with protein D by cloning cDNA into λKM10 vector. λKM8 and λKM10 are derivatives of λKM4 vector [6] obtained by introducing a flexible GS-linker between the displayed protein and gpD (Fig. 1). The insert size in the majority of the clones in the libraries ranged from 100 to 400 bp (Fig. 2). Only a tiny fraction of out-of-frame clones of this length do not contain stop codons, and are therefore displayed in the libraries constructed as N-terminus fusions, thus greatly reducing the probability of the selection of mimotopes.

### Selection of tumor-associated antigens

**Figure 3:**
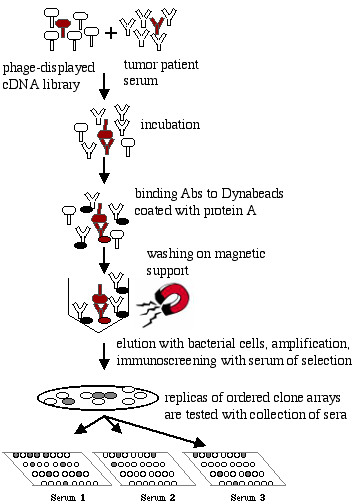
**Scheme of selection strategy leading to TAA identification. **A phage-displayed tumor cDNA library is preincubated with patient serum. TAA-specific antibodies bind to antigens exposed on the phage surface. Abs-phage complex is captured by protein A-coated solid support (ELISA plate or dynebeads). Non-bound phage are washed away. Bound phage are eluted by infection of added bacteria and amplified. Positive clones are isolated by immunoscreening procedure and then picked in ordered array on a bacterial lawn, transferred to nitrocellulose membrane and probed with different positive and negative sera.

The scheme of TAA identification is shown in Figure 3. Typically, one or two rounds of biopanning, performed according to the selection protocol described in Materials and Methods, were sufficient to obtain 2–50% of positive clones in the following immunoscreening procedure. Then, the identified phage clones were tested with a panel of positive and negative human sera by picking the clones in arrayed order on the bacterial lawn, blotting onto nitrocellulose membrane and probing with a number of different sera as previously described [6]. The nucleotide sequences of 21 clones that exhibited specific or preferential reactivity with sera from breast tumor patients as compared to sera from healthy donors were identified, and their nucleotide sequences were determined (Table 2 [see Additional file 1]).

### Serological analysis of tumor antigens

Phage lysates were prepared from all the selected clones as previously described [6] and tested in ELISA first with a collection of negative, and subsequently, with positive sera (Table 2 [see Additional file 1]). All the antigens tested reacted exclusively or preferentially with sera from breast cancer patients. Eight of the antigens reacted only with the patient serum used in the corresponding selection. Five antigens had cancer-related profile of reactivity, *P *< 0.05 (T6-2, T6-7, T7-1, T9-21 and T9-27). The other antigens either reacted with a low percentage of cancer sera, or the total panel of the tested sera was too small to offer any clear conclusion.

### Sequence analysis of selected cDNA clones

**Figure 4:**
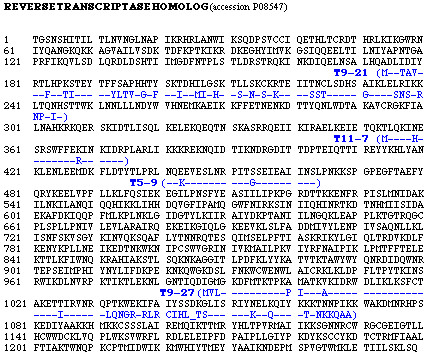
**Four identified antigen sequences with partial homology to reverse transcriptase homolog. **Peptide sequence is reported in single-letter code. Identical amino acids in the selected clones and reverse transcriptase homolog are represented by a dash. These clones were isolated from libraries of different origin. Clones T9-21 and T9-27, isolated from solid tumor library, had significantly high frequency of reactivity with sera from breast cancer patients.

Twenty-one positive clones were found to encode fragments from 18 different gene products, such as 4 clones (T5-9, T9-21, T9-27, T11-7) showing homology to different regions of the same reverse transcriptase gene (Figure 4). Most of the clones correspond to known gene products in the correct orientation and reading frame, with the exception of clone T5-18 encoding myc oncogen in an alternative frame. Several of these known gene products, such as reverse transcriptase homolog (clones T5-9, T9-21, T9-27, T11-7), protein kinase C-binding protein (T6-1), trap ankyrin repeat (T11-3), heat shock protein apg-2 (T11-13), have been previously identified by SEREX [9,14-17]. Eight of the sequences listed in Table 2 [see Additional file 1] encode for proteins with unknown functions.

### Cancer-specific expression of selected tumor antigens

**Figure 5:**
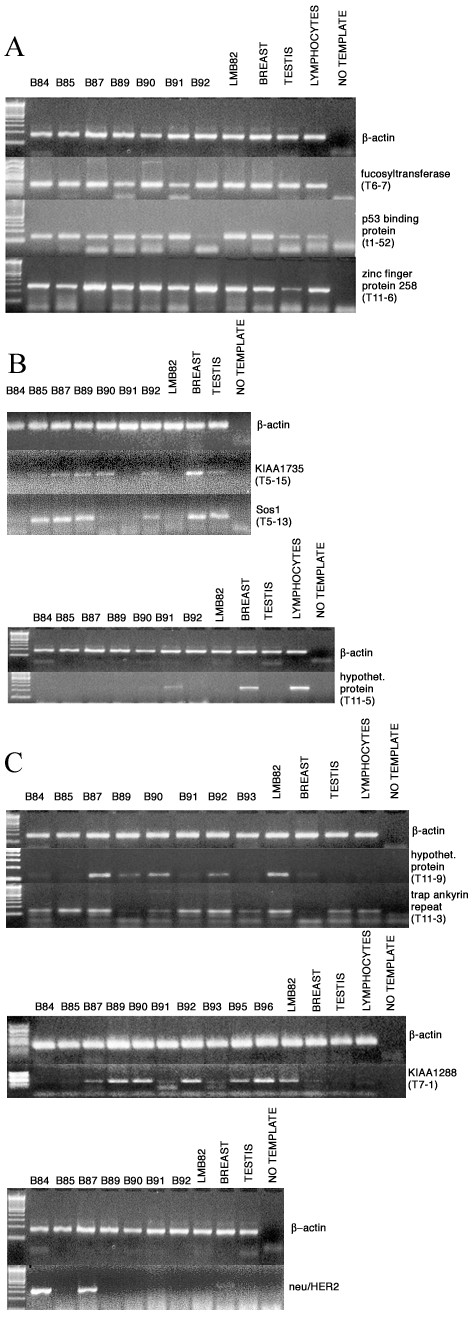
**cDNA-PCR analysis of gene expression was done using specific sequence primers. **We used SMART cDNAs from 7–10 different tumor samples (patients B84, B85, B87, B89, B90, B91, B92, B93, B95, B96) as template, from single metastasized lymph node indicated as LMB82 (patient B82) and from normal breast, normal testis, lymphocytes from healthy donors. cDNAs were normalized by amplification of β-actin gene. There are agarose gels with ubiquitously-expressed genes in Figure 5A, underexpressed genes in Figure 5B, overexpressed ones in Figure 5C.

Expression patterns for several of the selected genes were analyzed by semi-quantitative PCR from SMART cDNA template. It has been previously shown [18], by comparing the expression level of target genes in SMART PCR-amplified cDNAs and their corresponding total RNAs, that SMART cDNA accurately reflects gene expression patterns found in total RNA. We normalized the panel of cDNAs from ten different breast carcinomas, one metastasized lymph node, normal breast, normal testis and peripheral blood lymphocytes from healthy donors, by PCR amplification of a housekeeping gene, β-actin (Figure 5). Three of the identified antigens, fucosyltransferase (T6-7), Zinc finger protein 258 (T11-6), and p53-binding protein (T1-52) [6], were ubiquitously expressed in all the tumor and normal tissue samples tested (Figure 5A). Some of the antigens, T5-15 (KIAA1735), T5-13 (Sos1), T11-5 (hypothetical protein MGC4170) were found to be downregulated in many tumors (Figure 5B). T11-9 (hypothetical protein AF225417) was overexpressed in 50% of the primary tumors and the unique metastasized lymph node tested. T11-3 (trap ankyrin repeat) was overexpressed in most of the tumors tested in comparison with normal breast, although it was also transcribed in testis and normal lymphocytes (Figure 5C). T7-1 (KIAA1288) was found to be overexpressed in 50% of the primary breast carcinomas and in the metastasis specimen tested. In order to obtain an evaluation of the accuracy of the method used for the analysis of gene expression, we performed PCR amplification of neu/HER2, a known tumor marker overexpressed in breast cancer. We observed that neu/HER2 is overexpressed in 2 primary tumors among the 7 tested (≈29%) in accordance with the literature on breast carcinoma [19,20].

## Discussion

In the present study we report the construction of MCF-7 and MDA-MB-468 cell lines, breast carcinoma and testis cDNA phage-displayed libraries expressed as fusions to bacteriophage lambda gpD. The new phage vectors bear a flexible GS linker between the cloned protein domain and protein D, so as to facilitate lambda head assembly. Moreover, a new efficient protocol to synthesize cDNA was applied. We primed cDNA synthesis on mRNA template with random oligonucleotides containing a constant 5' end. After complete synthesis of double-stranded cDNA, a second round of random priming was applied to generate oriented fragments of cDNA suitable for library construction. This protocol, in comparison with previous version, increases the presence of authentic protein domains in the library twofold, because of correct cDNA orientation. Moreover, some of the clones isolated from our previous libraries were results of chimerical fusion of two or more different genes, generated through double random priming on ds cDNA template. The new protocol has reduced this problem significantly.

We also confirmed the advantage of N-terminal fusion for domain library construction in phage display vectors for screening with sera, because a significant amount of false positive cross-reactive clones, containing stop codons downstream of the fusion site giving rise to short mimotope sequences, were selected from the C-terminal fusion library (T6). Only 4 clones with specific tumor-related reactivity were isolated from the T6 library. However, C-terminal fusion might allow efficient display and selection for some antigenic C-terminal protein domains. In fact, the C-terminal fragment of fucosyltransferase (clone T6-7) was isolated from the T6 library.

The panel of selected TAAs in Table 2 [see Additional file 1] contains several functionally defined gene products, previously unknown as tumor antigens. AKAP450 and Sos1 proteins, corresponding to clones T5-8 and T5-13, are intracellular components of the signal transduction pathway. Sos1 is a well-known guanine nucleotide exchange factor for Ras oncogene [21]. Transgenic mice expressing a dominant form of Sos in basal keratinocytes develop skin papillomas with 100% penetrance [22]. Moreover, a Sos1 mutant, lacking four functionally important proline-rich (SH3 binding) regions was reported to be responsible for gingival fibromatosis [23]. AKAP450 is a member of the A-kinase anchor proteins family. It is located in the centrosome [24], and acts as a microtubule nucleation site [25] and as a scaffold for proteins involved in mitotic process [26].

Other selected antigens with known or predicted intracellular location are alpha-6-fucosyltransferase (clone T6-7) and zinc finger protein 258 (ZNF258, clone T11-6). Alpha-fucosyltransferase catalyzes the transfer of GDP-fucose to oligosaccharide chains linked to proteins, lipids and sugars [27] and resides in the luminal compartment of trans-Golgi vesicles [28]. The predicted protein product ZNF258 contains zinc-binding motif repeats [29]. If ZNF258, together with structural homology, also shares biological properties with zinc finger proteins, thus recognizing and interacting with DNA, it should have a nuclear localization. The presence on our antigen list of proteins with predicted intracellular residence is in agreement with findings from the other groups [30,5] and is related to possible tissue necrosis and cell lysis associated with tumor growth.

The human myc oncogene is transcribed from four alternative promoters giving rise to mRNAs for Myc1, Myc2, MYCHEX1 and 5'ORF [31]. Clone T5-18 is the result of the translation of an alternative frame to Myc1, Myc2 and does not correspond to any known protein product of myc oncogene transcription. It is not clear whether selection of this clone is an artifact of the experiment, or the result of an aberrant genome rearrangement in the tumor cells used for library construction.

Among isolated antigens, there are 4 clones (T5-9, T9-21, T9-27 and T11-7) having between 55–91% sequence identity with that of a reverse transcriptase homolog (Figure 4). Viral antigens corresponding to human endogenous retrovirus were previously isolated from renal cancers and melanomas by SEREX [9]. It is interesting to note that all these clones, isolated with sera from breast cancer patients, derive from libraries constructed with cDNA from every different origin utilized: i.e. cell lines (T5), solid tumor (T9), testis (T11). We have no explanation for the transcription of reverse transcriptase gene in normal testis tissue.

Eight proteins in the tumor antigen panel are unknown, or hypothetical proteins with unknown functions (T5-2, T5-15, T5-19, T6-2, T6-6, T7-1, T11-5, T11-9). The four underlined gene products from the list in parenthesis were analyzed for mRNA expression in tumors and normal breast tissue. The mRNA expression levels were analyzed by PCR from SMART-cDNA template in 7–10 breast cancer specimens, one metastasized lymph node, normal breast, testis and peripheral lymphocytes from healthy donors. Two of these 4 unknown antigens and T11-3 were found to be frequently overexpressed in breast cancer. In particular clone T7-1, which was classified as encoding for an unknown protein since it has 100% identity only with KIAA1288 from EST database, was found to be overexpressed in breast carcinomas. This finding, together with the good reactivity of T7-1 protein with sera from tumor patients, identifies this antigen among the most promising targets for diagnosis of the disease.

In contrast to the other antigens, which are overexpressed in breast cancer, mRNAs of T5-13, T5-15 and T11-5 appear to be underexpressed in 50–90% of breast cancer specimens, in comparison with normal breast tissue. How the immune system succeeds in responding to such antigens is still not clear. However, this finding is common to several SEREX-defined antigens, such as LU-12 [32], REN-9, REN-10 [33] and BR-41 [15], representing a group of TAAs deleted or downregulated in tumors. Lu-12, REN-9, Ren-10 map within cancer tumor suppressor gene locus at chromosome 3p21.3, a region often deleted in small cell lung cancer as well as in renal cancer. Downregulated antigen BR-41 was identified as SNT-1, a membrane-associated adaptor protein interacting with Sos1 [15]. In the present work we show that Sos1 (T5-13) is also downregulated in 50% of breast cancer samples. The downregulated antigens T11-5, T5-13 (Sos1), and T5-15 do not react with sera from patients B82-B96 analyzed for tumor mRNA expression. Furthermore, tumor biopsies from patients with good response for these antigens were not available for expression analysis. Thus, at present, it is not possible to determine whether T11-5, T5-13 (Sos1), and T5-15 are normally expressed, or downregulated, in patients showing an immune response for the corresponding antigen.

Sequence comparison of T11-5 and T5-15 clones with the EST database revealed identity with the hypothetical proteins MGC4170 and KIAA1735. We have derived the aa sequence of the corresponding ORFs and predicted the whole sequence architecture by computer analysis using the SMART program [34,35]). MGC4170 encodes for two NL domains, while KIAA1335 encodes for a 389 aa protein bearing a DIX domain at the carboxy-terminus. The presence of such structural domains indicates that both of these still unknown proteins (corresponding to clones T11-5 and T5-15), which we found downregulated in several tumor specimens, may be involved in the signal transduction machinery.

In spite of the fact that several promising antigens were identified from cDNA library constructed from testis mRNA, none of the antigens derived from testis or other libraries could be classified as specific testis/cancer (CT) antigen, because of their low expression in testis (T11-9, T7-1) or expression in other tissues as well.

In this work we analyzed the frequency of the immune response to the 21 identified antigens by using a panel of sera from tumor patients and healthy donors. In general, we observed a low frequency of serum reactivity with the antigens, which was expected and is similar to that of the vast majority of SEREX-identified clones [36]. A significant number of sera from tumor patients, in comparison with healthy individuals, efficiently recognized five of the identified antigens (T6-2, T6-7, T7-1, T9-21, T9-27). Clones T9-21 and T9-27, isolated from breast carcinoma library, respectively show 70% homology (55% identity) and 62% homology (68% identity) to reverse transcriptase homolog (PO8547). T7-1 is a protein having an unknown function, which was found to be overexpressed in breast carcinoma.

Taken together, these results lead us to believe that analysis of a complex panel of serologically-defined TAAs, with very large panels of sera from patients classified according to clinical parameters, i.e., age of patient, stage, extent and outcome of disease, etc. could lead to a much clearer understanding of the role, specificity and significance of the immune response versus disease in cancer patients.

## Conclusions

We demonstrated that a lambda display-based approach permits the efficient identification of tumor antigens, potential immunological targets in breast cancer. The list of 21 antigens identified in this work contains eight proteins of still unknown function. Three of the genes (T7-1, T11-3, T11-9) were found to be overexpressed in tumors as compared to normal breast. Five of the selected antigens (T6-2, T6-7, T7-1, T9-21, T9-27) were recognized specifically by breast cancer patient sera.

## List of abbreviations

aa, amino acids; Ag, antigen; EST, expressed sequence tags; ds cDNA, double-stranded cDNA; SEREX, serological identification of antigens by recombinant expression cloning; PAGE, polyacrylamide gel electrophoresis; PEG, polyethylene glycol; pfu, plaque-forming units; Sos1, son of sevenless homolog 1; TAA, tumor-associated antigen.

## Competing interests

EP, PV, AP, GM, EB, FF and OM are salaried by an organization holding two patents relating to the content of the manuscript.

## Authors' contributions

EP and PV contributed equally to the work. EP, PV and AP carried out cDNA library experiments, recombinant protein production, immunoassays and database search. GM and EB performed selection experiments. SB, MLD and MC contributed to immunological analysis of tumor antigens. ADPC and AL performed clinical studies. EC coordinated all medical aspects of the work. OM planned and performed molecular biology experiments, and teamed with FF in design and coordination of the entire project. All authors read and approved the final manuscript.

## Pre-publication history

The pre-publication history for this paper can be accessed here:



## Supplementary Material

Additional File 1 Table 2. List of identified TAAs. The File is given in Microsoft Word format. The table contains information about selected antigens. Click here for file

## Figures and Tables

**Table 1:** Lambda display libraries list.

Library name	Point of fusion with gpD	Source of cDNA	Library complexity
T5	N-terminus	MCF-7 + MDA-MB-468	1.7 × 10^7^
T6	C-terminus	MCF-7 + MDA-MB-469	3.4 × 10^7^
T7	N-terminus	Human breast carcinoma (patient B81)	1.5 × 10^6^
T9	N-terminus	Human breast carcinoma (patient B84)	2.3 × 10^7^
T11	N-terminus	Human testis	1.3 × 10^7^
