# Supplementary material for: Identification of a panel of tumor-associated antigens from breast carcinoma cell lines, solid tumors and testis cDNA libraries displayed on lambda phage
Source: BMC Cancer. 2004 Nov 12;4:78. doi: 10.1186/1471-2407-4-78 (PMC539249; doi:10.1186/1471-2407-4-78)
Supplement: Supplementary file 1 [file 1471-2407-4-78-S1.doc]

Table 2. List of identified TAAs. The P value is the probability that observed frequency distributions of reactivities of positive and negative sera are statistically the same according to the chi square test.

	Clones	Serum used for selection and immunoscreening	Length (aa)	Identity (accession number)	Classification	Frequency of phage clone reactivity		
						Pos. sera	Neg. sera	*P*
1	T5-2	B9	35	human genom DNA (AL589935)	-	1/15	0/15	0.31
2	T5-8	B20	83	AKAP protein (NP_671700)	unknown as TA	1/30	0/32	0.313
3	T5-9	B8	26	reverse trascriptase related protein (P08547)	TA [9]	4/35	1/38	0.138
4	T5-13	B55	132	Sos1 (NP_005624)	unknown as TA	2/60	0/52	0.184
5	T5-15	B72	100	KIAA1735	unknown as TA	7/60	1/43	0.081
6	T5-18	B17	56	myc oncogene (out of frame) (NM_002467)	unknown as TA	2/12	0/12	0.14
7	T5-19	B17	24	EST (AI903226)	unknown as TA	8/37	4/38	0.19
8	T5-20	B69	130	VRK1(vaccinia-related kinase 1) (NP_003375)	unknown as TA	3/40	0/24	0.169
9	T6-1	B9	64	protein kinase C-binding protein (Q9ULU4)	lymphoma TA [14]	6/36	0/12	0.131
10	T6-2	B9	15	not found	-	16/36	4/24	0.026
11	T6-6	B55	40	similar to MGC9515 protein (XP_351049)	unknown as TA	1/11	0/11	0.306
12	T6-7	B9	29	alpha 1,6-fucosyltransferase (NP_835370)	unknown as TA	7/28	1/24	0.025
13	T7-1	B81	75	KIAA1288/NEP (AAQ24172)	unknown as TA	18/40	4/24	0.021
14	T9-21	B84	73	reverse trascriptase related protein (P08547)	TA [9]	6/16	0/14	0.011
15	T9-27	B15	82	reverse trascriptase related protein (P08547)	TA [9]	6/31	0/32	0.009
16	T11-3	B57	71	trap ankyrin repeat/ NY-BR-16 (NP_115593)	TA [15]	2/25	0/12	0.314
17	T11-5	B16	71	MGC4170 protein (NP_077288)	unknown as TA	1/18	0/20	0.285
18	T11-6	B81	96	Zinc finger protein 258 (NP_009098)	unknown as TA	1/20	0/20	0.311
19	T11-7	B81	22	reverse trascriptase related protein (P08547)	TA [9]	1/20	0/20	0.302
20	T11-9	B81	82	hypothetical human protein (AF225417)	unknown as TA	1/11	0/9	0.353
21	T11-13	B84	81	heat shock protein apg-2 (P34932)	TA [16, 17]	1/15	0/14	0.326
